# Preeminence of Lesser Splanchnic Blood Flow in Selected Patients With Generalized Portal Hypertension

**DOI:** 10.1155/1990/94864

**Published:** 1990

**Authors:** Charles L. Witte, Marlys H. Witte, Gerald D. Pond

**Affiliations:** 1 Departments of Surgery and Diagnostic Radiology University of Arizona College of Medicine Tucson Arizona 85724 USA; 2 University of Arizona College of Medicine 1501 N. Campbell Avenue Tucson AZ 85724 USA

## Abstract

Although restricted transhepatic portal flow is necessary for development of generalized portal hypertension (GPH), increased splanchnic arterial inflow also contributes to GPH and its clinical sequelae. In this context, we describe 7 male and 6 female patients (mean age 48 years) in whom the lesser splanchnic (gastrosplenic) system played a key role in the signs and symptoms of GPH. These 13 patients (9 with hepatic cirrhosis, 3 with primary myeloproliferative disorder, and 1 with extrahepatic portal block) shared common features of massive splenomegaly, huge splenofundic gastric varices, often with a prominent natural shunt to the left renal vein. Total or near total splenectomy alone or combined where appropriate with coronary vein ligation was effective in controlling varix hemorrhage (10 patients), ascites (3), or complications of an enlarged spleen-anorexia and abdominal pain (3), hemolytic anemia (1) and profound thrombocytopenia with severe epistaxis (1). Intraoperative jejunal portal venography was crucial in operative management in order to establish definitively the presence or absence of coronary venous collaterals, and when present, to verify their operative ligation.

These distinctive patients illustrate: 1) GPH is a heterogeneous syndrome of divergent splanchnic circulatory patterns, a feature which should be taken into account in selecting operative treatment; 2) one well-defined subgroup displays prominent hyperdynamic lesser splanchnic and specifically, splenic blood flow as a major contributor to clinical complications; and 3) within this subgroup, splenectomy combined with documented absence or surgical interruption of coronary venous collaterals as corroborated by intraoperative portography is effective alternative treatment.


*HPB Surgery*, 1990, Vol. 2, pp. 233–251
Reprints available directly from the publisher
Photocopying permitted by license only

© 1990 Harwood Academic Publishers GmbH
Printed in the United Kingdom

### PREEMINENCE OF LESSER SPLANCHNIC BLOOD FLOW IN SELECTED PATIENTS WITH GENERALIZED PORTAL HYPERTENSION

CHARLES L. WITTE, MARLYS H. WITTE, GERALD D. POND
*Departments of Surgery and Diagnostic Radiology, University of Arizona College of Medicine, Tucson, Arizona 85724*

*(Received 12 December 1989)*

Although restricted transhepatic portal flow is necessary for development of generalized portal hypertension (GPH), increased splanchnic arterial inflow also contributes to GPH and its clinical sequelae. In this context, we describe 7 male and 6 female patients (mean age 48 years) in whom the lesser splanchnic (gastrosplenic) system played a key role in the signs and symptoms of GPH. These 13 patients (9 with hepatic cirrhosis, 3 with primary myeloproliferative disorder, and 1 with extrahepatic portal block) shared common features of massive splenomegaly, huge splenofundic gastric varices, often with a prominent natural shunt to the left renal vein. Total or near total splenectomy alone or combined where appropriate with coronary vein ligation was effective in controlling varix hemorrhage (10 patients), ascites (3), or complications of an enlarged spleen-anorexia and abdominal pain (3), hemolytic anemia (1) and profound thrombocytopenia with severe epistaxis (1). Intraoperative jejunal portal venography was crucial in operative management in order to establish definitively the presence or absence of coronary venous collaterals, and when present, to verify their operative ligation.

These distinctive patients illustrate: 1) GPH is a heterogeneous syndrome of divergent splanchnic circulatory patterns, a feature which should be taken into account in selecting operative treatment; 2) one well-defined subgroup displays prominent hyperdynamic lesser splanchnic and specifically, splenic blood flow as a major contributor to clinical complications; and 3) within this subgroup, splenectomy combined with documented absence or surgical interruption of coronary venous collaterals as corroborated by intraoperative portography is effective alternative treatment.

KEY WORDS: Portal hyptertension, splenic blood flow, splenectomy, intraoperative portography

Although splenomegaly of liver disease is associated with hyperdynamic blood flow, the role of the spleen and the lesser splanchnic circulation in the pathogenesis of varix hemorrhage and ascites is usually deemed of secondary importance[1]. In hepatic cirrhosis, devascularization by splenectomy alone or in combination with interruption of gastric arteries commonly fails to halt varix bleeding[2] either because splenic blood flow is only a minor factor in the development of portal hypertension, or because augmented intestinal blood flow continues unchecked to the esophago-gastric venous plexus via greater splanchnic (i.e.mesenteric-gastric or coronary) venous collaterals. A notable exception is varix hemorrhage with left-sided or lesser splanchnic (gastrosplenic) portal hypertension due to segmental obstruction to the splenic vein from thrombosis or stricture. In this condition, in contrast to

Correspondence to: Charles L. Witte, M.D. Professor of Surgery, University of Arizona College of Medicine, 1501 N. Campbell Avenue, Tucson, AZ 85724

Support in part by the Arizona Disease Control Research Commission Contract #8277-000000-1-0-WE-9313, Phoenix, Arizona generalized portal hypertension, transhepatic portal flow is unrestricted and interruption of arterial inflow by splenectomy alone is sufficient treatment[3].

As with varix hemorrhage, the lesser splanchnic portal system is also rarely, if ever, considered a contributing factor to the pathogenesis of ascites; accordingly, splenectomy is seldom recommended. Even pancytopenia in portal hypertension, which is a direct consequence of hypersplenic trapping and accelerated destruction of peripheral blood elements, is usually considered an insufficient reason for splenectomy[4], and if advocated for profound thrombocytopenia and bleeding, concomitant portasystemic decompression (e.g. splenorenal shunt) is also usually advised[5].

Despite these generally accepted tenets, patients with generalized portal hypertension display a heterogeneous spectrum of visceral lymphatic and blood circulatory patterns[6]. Based on these different splanchnic variations along with divergent clinical presentations, we earlier suggested[7] that:

*"In considering the syndrome of portal hypertension, it is inappropriate to argue abstractly whether portal flow is increased, decreased or reversed, whether splenectomy is 'good' or 'bad' treatment for ascites or varix hemorrhage, whether enhancing portal venous outflow or decreasing arterial inflow is 'better' treatment. Instead, in each patient...blood and lymph circulatory dynamics should be delineated insofar as necessary to design appropriate treatment."*

With time we have garnered further evidence supporting this view, and we now define a subgroup of patients with generalized portal hypertension in whom the lesser splanchnic (gastrosplenic) circulation is an important contributor to varix hemorrhage, ascites, and severe peripheral cytopenia. In this patient subpopulation, preoperative arterial portography and intraoperative portal venography have proved indispensable for optimizing surgical management.

### *Case Studies*

This report is composed of 13 selected patients (7 male and 6 female) with generalized portal hypertension. The age range was from 8 to 70 years (mean 48 years). The presenting signs or symptoms included one or more of the following: hematemesis from varices (9 patients), ascites (3), anorexia and abdominal pain (3), hemolytic anemia (1), and epistaxis (1). Hepatic cirrhosis was the underlying cause of portal hypertension in 9 patients. Three patients had a myeloproliferative disorder and one cavernous transformation of the portal vein (see Table 1).

### *Hepatic Cirrhosis*

Case 1. A 55-year-old alcoholic man with external stigmata of liver disease developed the acute onset of hematemesis. Chemical tests of liver function were deranged including a modestly elevated prothrombin time and modest bilirubinemia. Gastroesophageal endoscopy on two occasions failed to reveal a specific site of bleeding and of note, varices were not seen. Digital-subtraction visceral arterioportography demonstrated a massively enlarged spleen with large gastric fundic varices arising solely from the splenic vein (Figures 1A and 1B). Because of persistent gastric bleeding. laparotomy was undertaken which confirmed micronodular cirrhosis and splenomegaly. Ascites was absent. Cannulation of a jejunal mesenteric vein yielded a portal pressure of 35cm saline and intraoperative

**Table 1** Summary of Clinical Findings

| | *Age (yrs)* | *Sex M/F* | *Chief Sign or Symptom* | *Major Diagnosis* | *Key Portogram Features* | *Treatment* | *Spleen Weight gms* | *Outcome* |
|---|---|---|---|---|---|---|---|---|
| 1. | 55 | M | Hematemesis | Cirrhosis (alcoholism) | Spleno-fundic varices No C.V. | Splenectomy | 1100 | Well (6 months, lost to follow-up) |
| 2. | 17 | M | Hematemesis | Cirrhosis (cystic fibrosis) | Spleno-fundic varices spontaneous fundic-renal shunt. Small C.V. | Splenectomy ligation C.V. | 1250 | Well (20 Months) |
| 3. | 48 | M | Hematemesis | Cirrhosis (hypopituitarism) | No C.V. | Splenectomy* | 850* | Well ($3\frac{1}{4}$ years) |
| 4. | 10 | M | Anorexia, Abd. pain Hematemesis | Cirrhosis (methotrexate histiocyosis-X) | Spleno-fundic varices moderate C.V. | Splenectomy* ligation C.V. | 600* | Well ($6\frac{3}{4}$ years) |
| 5. | 47 | F | Hematemesis | Cirrhosis (post-hepatitis) | Spleno-fundic varices Spontaneous fundic-renal shunt | Splenectomy ligation C.V. gastric wall stapling | 900 | Sudden death (4 weeks) (Autopsy pulmonary micro-embolism) |
| 6. | 41 | F | Ascites | Cirrhosis (cryptogenic) | Marked portal-splenic ectasia. No E-G varices | Splenectomy | 1100 | Dead (12 years) Renal failure |
| 7. | 51 | F | Anorexia, pain Ascites | Cirrhosis (post-hepatitis) | Spleno-fundic varices No C.V. | Splenectomy | 950 | Well (26 months) |
| 8. | 59 | F | Epistaxis anorexia Abd. pain | Cirrhosis (post-hepatitis) | Spleno-gastroesophageal varices | Splenectomy | 550 | Dead (7 years) fulminant pneumococcemia |
| 9. | 61 | M | Hematemesis | Cirrhosis (alcoholism) | Spleno-fundic varices spontaneous fundic-renal v. shunt, moderate C.V. | Splenectomy ligation C.V. gastric wall stapling | 1350 | Dead (3 weeks) hepato-renal failure |
| 10. | 67 | F | Hematemesis | CML | Spleno-fundic varices large C.V. | Splenectomy ligation C.V. | 1100 | Dead (1 year). Disseminated H. zoster |
| 11. | 57 | F | Hematemesis Ascites | Polycythemia vera | Marked portal-splenic venous-ectasa | Splenectomy | 4000 | CML (5 years) |
| 12. | 70 | M | Hemolysis | ? myeloid metaplasia | — | Splenectomy | 1200 | Dead (1 year) fulminant pneumococcemia |
| 13. | 39 | M | Hematemesis | Cavernous transformation p.v. | P.V. & S.M.V. obstruction Spleno-gastric varices. No C.V. | Splenectomy gastric wall stapling | — | Well (23 months) |

* A small remnant (approx. 50–80 gms) was left in-situ

C.V. = coronary vein; E-G = esophagogastric; pv = portal vein; smv = superior mesenteric vein; abd = abdominal; CML = chronic myelogenous leukemia

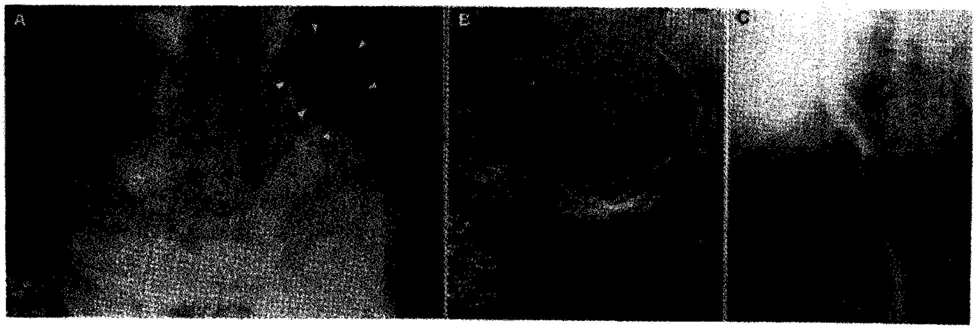

**Figure 1.** Digital-subtraction visceral arterial portography (A,B) and intraoperative jejunal venoportography (C) demonstrating absence of coronary venous tributaries and large gastric fundic varices (arrowheads) arising exclusively from the splenic (lesser splanchnic) circulation. (Patient 1).

portography (60ml of 60% Renografin, Squibb Diagnostics, New Brunswick, N.J.) revealed a patent portal vein and no mesenteric-gastric (coronary) venous collaterals (Figure 1C). After an anterior gastrotomy blood gushed from the fundus, and a racemose collection of "tumor varices" were rapidly oversewn following which splenectomy was done. In the course of removal of the spleen (weight 1100gm) giant short gastric veins (~2cm wide) emanating from the splenic hilum were obliterated. After a stormy postoperative course from pulmonary and hepatic dysfunction, he was discharged with no further bleeding. Six months later he was well but thereafter was lost to follow-up.

Case 2. A 17-year-old man with cystic fibrosis and hepatic cirrhosis developed the sudden onset of unremitting hematemesis. Esophagoscopy disclosed large gastro-fundic varices but no esophageal varices. Chemical tests of liver function were mildly deranged with a slightly elevated serum bilirubin. Splenic puncture yielded a pressure of 29cm saline and splenoportography displayed huge gastro-fundic varices originating almost entirely from the splenic hilum, a small coronary vein and variceal decompression via a spontaneous fundic left renal venous shunt (Figure 2A). Because of persistent gastric bleeding, laparotomy was undertaken which confirmed macronodular cirrhosis and massive splenomegaly. Ascites was absent. After splenectomy (spleen weighed 1250gm) and oversewing of huge greater curvature gastric varices draining from the splenic hilum, the jejunal venous pressure remained at 34cm saline. The coronary vein was also ligated. Intraoperative venography depicted decompression of residual fundic varices into the renal vein via a large retroperitoneal collateral shunt (Figure 2B). Twenty months later he is well; bleeding has not recurred.

Case 3. A 48-year-old man with congential hypopituitarism (Froelich's syndrome) suddenly developed hematemesis necessitating several transfusions. Physical examination disclosed a markedly enlarged spleen in addition to generalized feminization. Visceral arterial portography disclosed a paraumbilical collateral vein and "cork screwing" of the hepatic arteries consistent with cirrhosis and portal hypertension. At operation undertaken for profound thrombocytopenia and

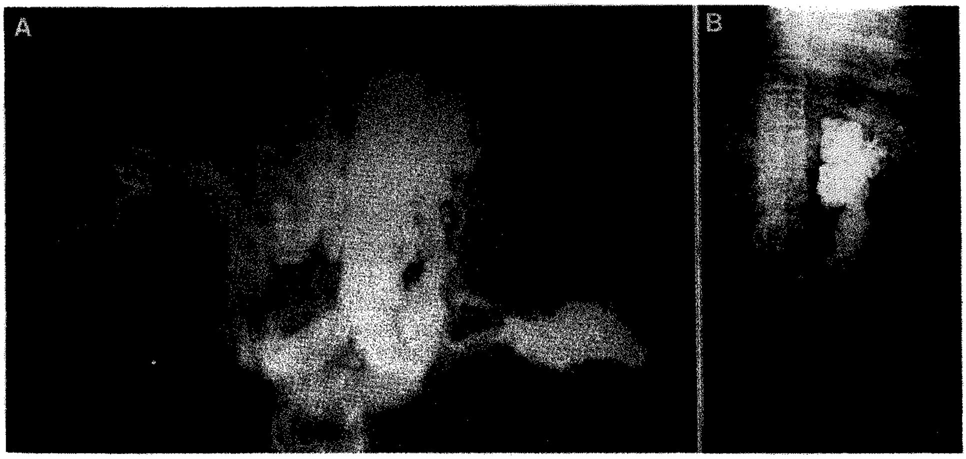

**Figure 2.** Splenoportogram (A) demonstrating gigantic gastro-fundic varices originating almost entirely from the splenic hilum, a comparatively small coronary vein and a natural fundic variceal-left renal venous shunt. After splenectomy and coronary vein ligation, portal venography (B) shows only a residual fundic variceal renal vein shunt. (Patient 2).

leukopenia ($30 \times 10^3/mm^3$ and $1.5 \times 10^3/mm^3$, respectively), the liver showed micronodular cirrhosis and the spleen was notably enlarged. Jejunal vein cannulation yielded a venous pressure of 33cm saline and operative portography showed a large paraumbilical vein but notably no collateralization from the mesenteric-portal (greater splanchnic) system to the esophagogastric junction. A subtotal splenectomy was performed (excised spleen weight 770gm) and a remnant spleen ($\sim$80gm) was left in situ attached to the highest vasa brevia. Three years and three months after operation the peripheral platelet and white blood counts are within normal limits and digestive tract hemorrhage has not recurred.

Case 4. An 8-year-old boy with stunted growth underwent distal splenorenal shunt for refractory bleeding of esophageal varices secondary to portal hypertension from methotrexate-induced hepatic fibrosis as a result of treatment for histiocytosis-X (malignant Langerhans histiocytosis). Two years later, left upper quadrant abdominal pain, substernal discomfort and early satiety accompanied by severe neutropenia and leukopenia prompted celiac angiography which disclosed occlusion of the shunt (Figure 3A). At operation cannulation of a jejunal mesenteric vein yielded a portal pressure of 28cm saline and portography demonstrated a single coronary vein draining to varices at the esophagogastric junction (Figure 3B). The spleen was markedly enlarged, engorged, and tense. A subtotal splenectomy (weight 600gm) was done leaving behind a remnant approximately 30gm attached to the highest short gastric vessels. The coronary vein was obliterated with vascular clips as corroborated by intraoperative portal venography (Figure 3C). Repeat jejunal venous pressure was 25cm saline. Over the ensuing 6 and 3/4 years the peripheral blood count has remained within normal limits even though the splenic remnant has grown. Hematemesis has not recurred.

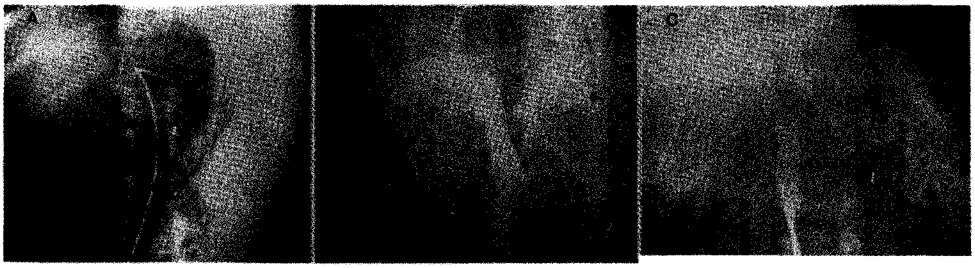

**Figure 3.** Splenic arterial portography (A) demonstrating massive splenomegaly and occlusion of a previously constructed distal-splenorenal shunt. Intraoperative portograms (B,C) via a jejunal mesenteric vein demonstrates a prominent coronary vein with fundic varices (B, arrows). After subtotal splenectomy and coronary vein ligation, contrast now preferentially fills retroperitoneal venous collaterals (C). (Patient 4).

Case 5. A 47-year-old woman with acquired panhypopituitarism (Sheehan's syndrome) and postnecrotic cirrhosis developed recurrent episodes of hematemesis. Physical examination disclosed prominent splenomegaly and gastroesophagoscopy confirmed large gastro-fundic and small esophageal varices. Visceral arterial portography confirmed massive fundic varices arising from the splenic hilum and partially "decompressing" via phrenic-adrenal collaterals into the left renal vein and inferior vena cava (Figure 4A). A small coronary vein was also seen (Figures 4A and 4B) Laparotomy confirmed micronodular cirrhosis and massive splenomegaly. Cannulation of a jejunal mesenteric vein yielded a portal pressure of "only" 22cm saline. The spleen was removed (weight 900gm), a small coronary vein was oversewn, and the anterior and posterior gastric wall just below the esophagogastric junction was separately stapled (TA-55) via a small gastrotomy in the greater curvature. Repeat jejunal venous pressure was 19cm saline and intraoperative portography showed no residual mesenteric-gastric (coronary) venous collaterals (Figure 4C). Despite removal of the spleen, a progressive and severe thrombocytopenia developed attributed to thrombotic thrombocytopenic purpura (TTP) and persisted until time of sudden death 4 weeks after operation. Digestive tract hemorrhage did not recur. Autopsy disclosed microembolic pulmonary hypertension and no untoward intraabdominal complication.

Case 6. A 41-year-old female with cryptogenic cirrhosis developed massive ascites refractory to dietary salt restriction and diuretic drugs (Figure 5). The spleen was markedly enlarged and extensive work-up of splanchnic blood and visceral lymph circulatory dynamics corroborated that splenic hyperdynamic blood flow was a major contributor to portal hypertension (for details see, refs.8,9) (Figure 5). After removal of the spleen (weight 1100gm) mesenteric venous pressure fell from 38 to 25cm saline. For the next 10 years she remained free of ascites (Figure 5) without supplemental drug therapy, but then developed end-stage renal failure, was placed on chronic hemodialysis, and ultimately died with anasarca 12 years after splenectomy.

Case 7. A 51-year-old woman with postnecrotic cirrhosis developed moderate ascites, early satiety, weight loss, and unremitting left upper quadrant abdominal

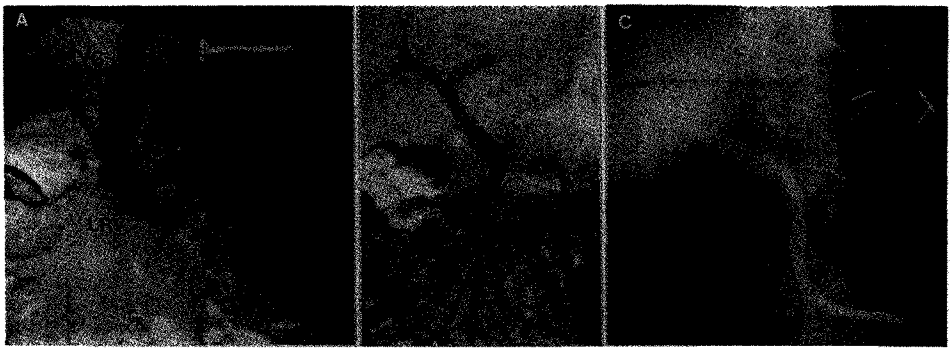

**Figure 4.** Visceral arterial portography (A,B) demonstrating massive fundic varices arising from the splenic hilum and partially decompressing into the left renal vein (LRV) and inferior vena cava (IVC). A small coronary venous contribution (arrows) is also seen. After splenectomy and coronary vein ligation, intraoperative portography corroborates interruption of the coronary vein and lack of filling of esophagastric varices (C). (Patient 5).

pain in conjunction with massive splenomegaly. Esophagogastroscopy disclosed moderate distal esophageal and gastrofundic varices while visceral arterial portography disclosed in addition to splenomegaly extensive venous collaterals to the esophagogastric junction arising from the splenic hilum. Laparotomy demonstrated moderate ascites, extensive edema of the small bowel and pancreas, dilated lymphatics throughout the small bowel mesentery as well as macronodular cirrhosis and massive splenomegaly. Cannulation of a jejunal mesenteric vein yielded a portal pressure of 35 cm saline and intraoperative portography revealed absence of mesenteric-gastric (coronary) collaterals. During splenectomy (spleen weight 950gm) huge (2cm) venous collaterals from the splenic hilum along the greater curvature of the stomach were ligated and divided. Repeat portal pressure was unchanged. Recovery from operation was uneventful and over the ensuing 26 months her appetite returned, weight increased, and symptoms of satiety disappeared. Moreover, she has been free of ascites and has had no episodes of hematemesis.

Case 8. A 59-year-old frail (<100 lbs) woman with longstanding postnecrotic cirrhosis, esophageal varices and intermittent ascites developed severe, intractable nose bleeds, easy bruisability, diffuse petechial eruption, increasing pigmentation of the legs, and early satiety. Peripheral blood counts and bone marrow aspirate were consistent with severe hypersplenism with bleeding times (Ivy) greater than 13 minutes (for details see ref.7). Superselective splenic arterial portography demonstrated a large tortuous splenic artery, massive splenomegaly, and huge esophagogastric varices arising primarily from the splenic circulation. After splenectomy (500gm) the jejunal mesenteric venous pressure fell from 29 to 22 cm saline. The clinical course over the next 5 years was unremarkable. Specifically, hypersplenism was corrected, ascites did not recur, hematemesis did not intercede, epistaxis was rare, and hyperpigmentation of the legs dramatically improved. Seven years later, however, she suddenly developed fulminant bacteremia from lobar pneumonia (S.pneumoniae) and died within 36 hrs despite high-dose penicillin therapy. She

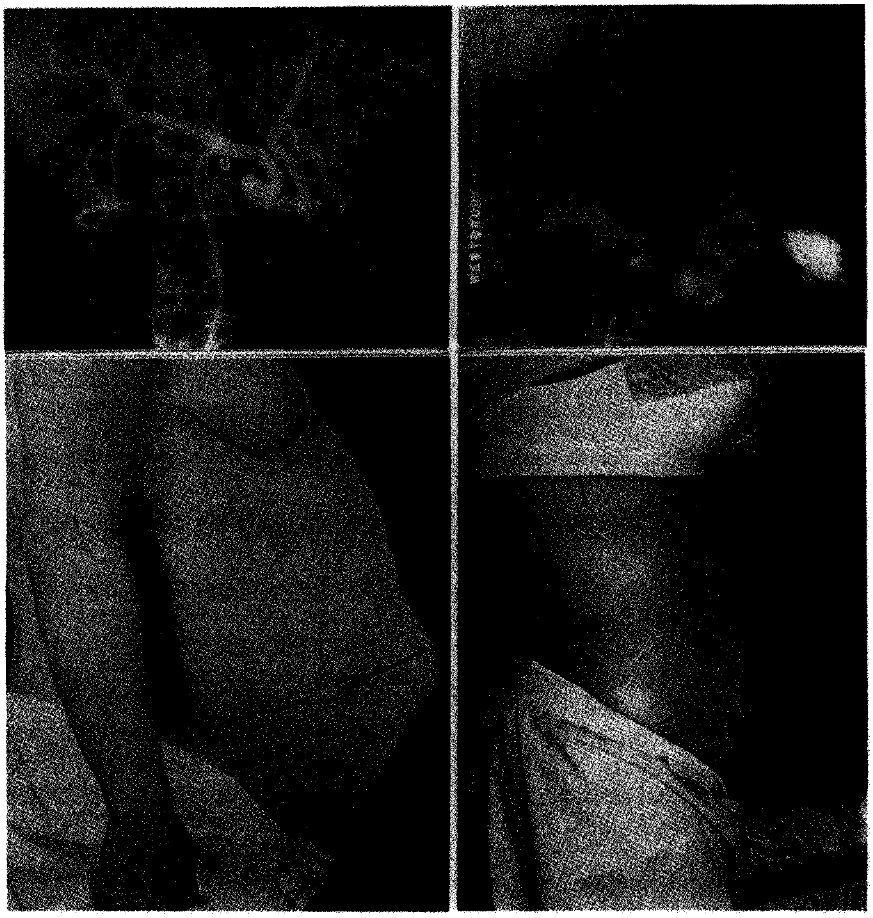

**Figure 5.** 41 year-old woman with intractable ascites (lower left) from hepatic cirrhosis. Celiac arteriography and splenoportography (upper frames) demonstrate a markedly enlarged splenic artery with porto-splenic venous ectasia. After splenectomy, portal venous pressure fell from 38 to 25 cm saline and ascites promptly remitted (lower right). (Patient 6).

had not received soluble pneumococcal vaccine nor were prophylactic antibiotics prescribed. Autopsy disclosed a large hepatoma of the left lobe of the liver.

Case 9. A 61-year-old man with longstanding alcohol abuse developed refractory hematemesis. Physical examination disclosed an enlarged spleen and "feminization" consistent with cirrhosis of the liver. Gastroesophagoscopy revealed large non-bleeding esophageal varices with copious clotted blood in the stomach. A limited attempt at endosclerotherapy was done. Superior mesenteric arterial portography showed on the venous phase retrograde flow into the splenic vein with a small coronary vein feeding varices into the esophagogastric junction (Figure 6A). Splenic arterial portography demonstrated massive splenomegaly with splenic blood flow diverted into huge fundic varices draining via a large retroperitoneal

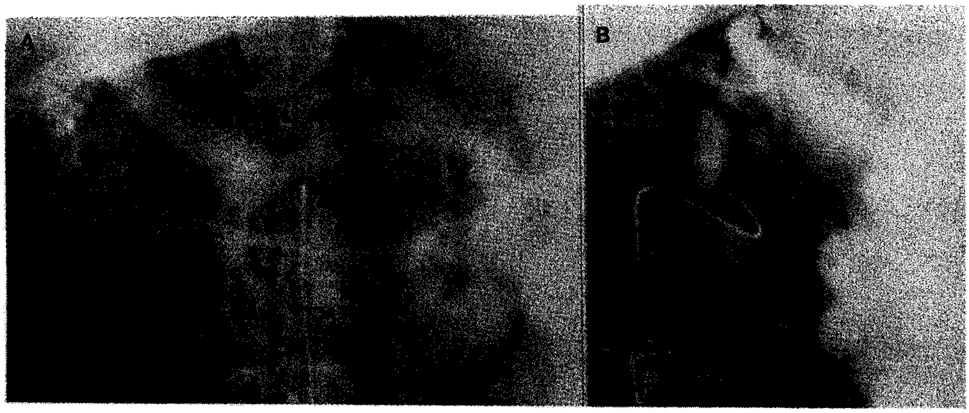

**Figure 6.** Superior mesenteric arterioportography (A) demonstrating retrograde filling of splenic vein and comparatively small coronary vein. Splenic arterial portogram (B) displays massive perisplenic hilar varices with a large natural retroperitoneal shunt. (Patient 9) (See Figure 8).

trunk into the left renal vein. Perisplenic varices were especially large (Figure 6B). Prompt laparotomy confirmed micronodular hepatic cirrhosis and massive splenomegaly. The stomach was markedly distended with 6 to 8 units of clotted blood; after evacuation of the gastric contents, splenectomy was undertaken (weight 1350gm). Giant parasplenic collateral veins in the vicinity of the spleen hilum and Gerota's fascia were oversewn. Jejunal vein cannulation yielded a portal pressure of 27cm saline which rose slightly after splenectomy. Intraoperative portal venography sharply demarcated mesenteric-esophageal (coronary) venous tributaries. These venous channels were "hemoclipped" until serial intraoperative portograms confirmed that the "feeders" were interrupted (Figures 7A-7C). Intermittent gastro-esophageal bleeding recurred sporadically for 4 days and was controlled by further transendoscopic sclerotherapy at which time small residual varices with post-sclerotherapeutic ulcerations were confirmed. Bleeding thereafter ceased but he died of progressive hepatic and renal failure 3 weeks after operation. At autopsy, portal venography via a jejunal vein confirmed that venous collaterals to the esophagogastric junction had been effectively interrupted (Figure 7D).

*Myeloproliferative Disorders*

Case 10. A 67-year-old woman with chronic myelogenous leukemia (white blood cell count 30–50,000/mm$^3$) on low-dose chlorambucil (4 mg/day) developed repeated episodes of hematemesis secondary to endoscopically verified esophagogastric varices. Physical examination was noteworthy only for a markedly enlarged spleen. Visceral arterial portography disclosed large gastric fundic varices emanating from both the lesser and greater splanchnic circulations (Figures 8A, 8C). At operation, in addition to massive splenomegaly (Figure 8B) the liver appeared "cirrhotic" but subsequent histopathology showed nodular regenerative hyperplasia. Intraoperative cannulation of a jejunal mesenteric vein yielded a venous pressure of 30cm saline which was unchanged after splenectomy (spleen weight

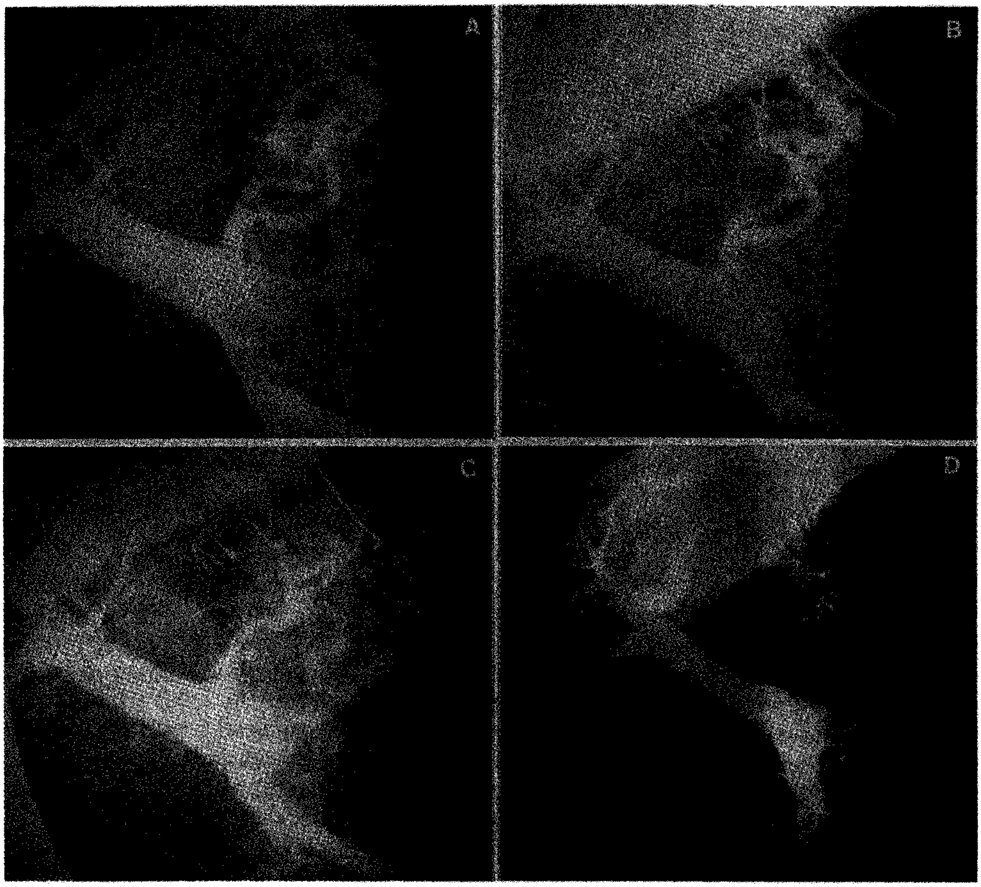

**Figure 7.** Intraoperative portography after splenectomy (patient 9), sharply demarcating coronary venous tributaries still filling esophageal varices (A). Ligation of coronary venous tributaries was at first only partially successful (B) but after several efforts repeat operative portogram demonstrated interruption of the esophagogastric varix filling (C). Repeat portography (D) at autopsy three weeks later from hepatorenal failure confirmed interruption of the coronary venous tributaries and lack of esophagogastric variceal communication.

1100gm). During the splenectomy, huge venous collaterals along the greater curvature of the stomach arising from the splenic hilum and with palpable thrill were carefully oversewn and "hemoclipped". The coronary vein was then ligated and its interruption corroborated by intraoperative portography (Figure 8D). The postoperative period was uneventful; however, 12 months later she developed disseminated herpes zoster infection and died. Gastrointestinal bleeding had not recurred.

Case 11. A 57-year-old woman with polycythemia vera developed prominent ascites, marked splenomegaly (Figure 9A), and bleeding of esophageal varices. Superselective splenic arterial portography demonstrated a markedly enlarged

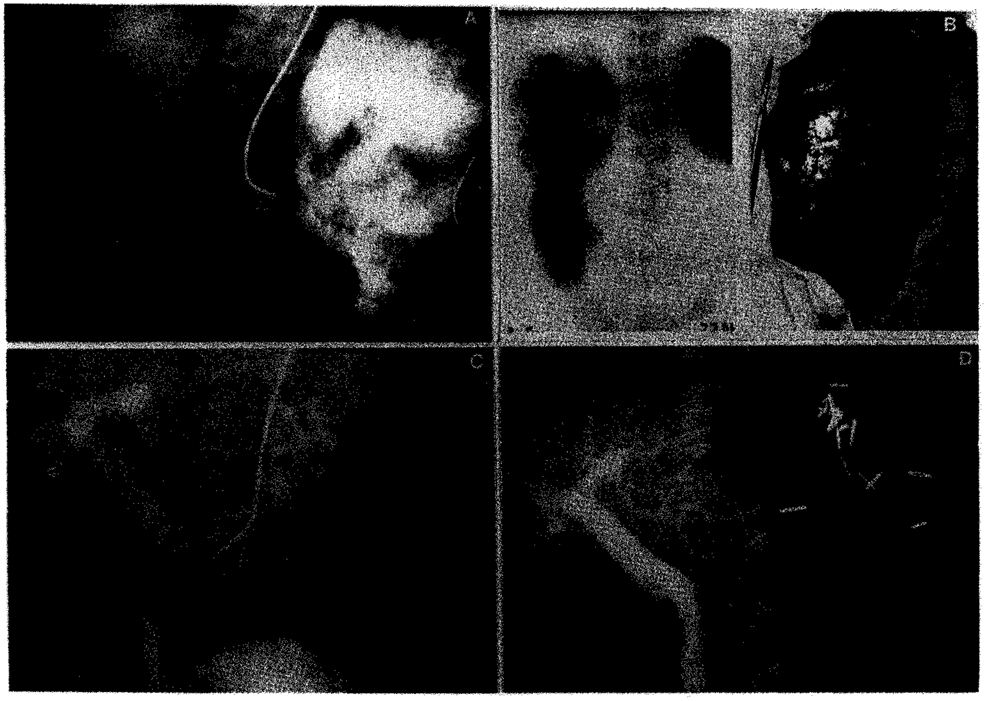

**Figure 8.** Visceral arterial portography (A,C) demonstrating massive gastric varices from both splenic (A) and mesenteric-portal (coronary) tributaries (C). At operation the spleen was massively enlarged and partially infarcted as seen preoperatively on technetium sulfur colloid scintigraphy (B). After splenectomy and coronary vein ligation, intraoperative portography (D) confirmed interruption of mesenteric-gastric venous collaterals and lack of esophagogastric varices. (Patient 10).

splenic artery with small polar aneurysms (Figure 9B). On the venous phase, a huge, tortuous splenic and portal vein were displayed (Figure 9C). At operation, the spleen was removed (4kg) and portal pressure (via a jejunal mesenteric venous catheter) fell from 36 to 26cm saline. Although the patient's disease several years later progressed to chronic myelogenous leukemia, she remained free of ascites and varix hemorrhage.

Case 12. A 70-year-old man developed hemolytic anemià necessitating increasingly frequent transfusions accompanied by marked splenomegaly, leukopenia, thrombocytopenia, and hypergammaglobulinemia. Splenectomy was undertaken (spleen weight 1200gm). At operation, jejunal mesenteric venous pressure was 35cm saline and fell to 23cm saline after splenectomy. Of note there were extensive venous collaterals in the left upper quadrant adjacent to the spleen and left hemidiaphragm. Although the postoperative recovery was unremarkable and the pancytopenia was corrected, he died 6 months later of fulminant pneumococcal pneumonia.

### *Extrahepatic Portal Block*

Case 13. A 39-year-old man with history of alcohol abuse developed bright red

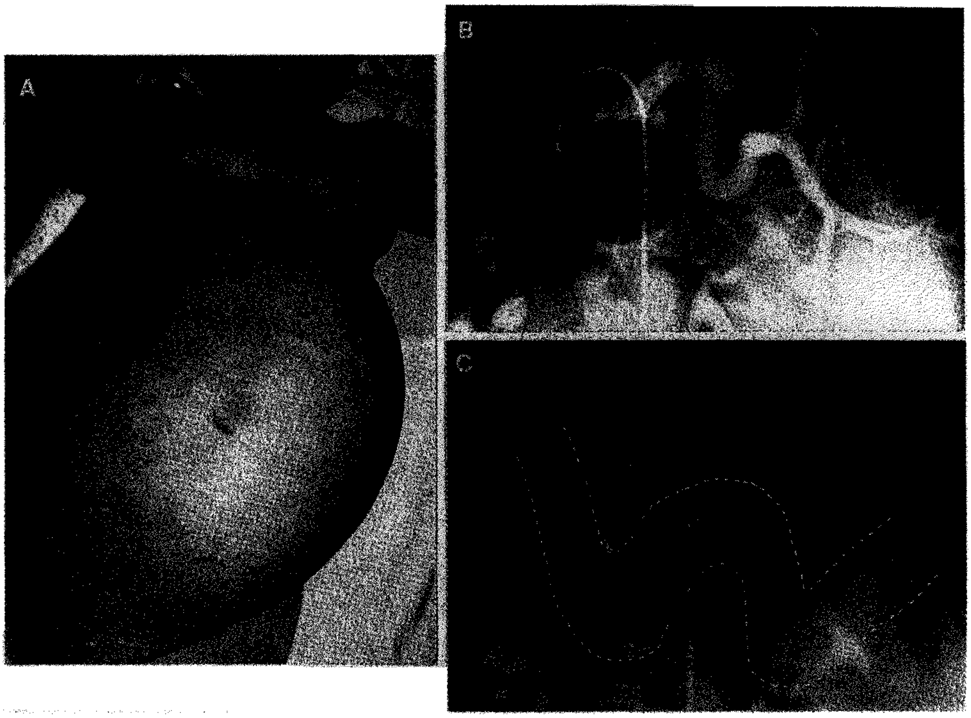

**Figure 9.** 51-year-old woman with massive ascites and bleeding of esophageal varices from a primary myeloproliferative disorder (A). Splenic arterial portography revealed marked enlargement of the splenic artery and a huge portal system. Removal of the spleen sharply decreased portal presure from 36 to 26 cm saline and led to remission of ascites and varix hemorrhage. (Patient 11).

emesis and gastroesophagoscopy disclosed distal esophageal and large gastro-fundic varices with copious maroon-colored blood in the stomach. Except for pallor and tachycardia, there were no notable findings on physical examination. Laboratory findings disclosed a white count of 2,500/mm$^3$ and a platelet count of 63,000/mm$^3$ while chemical tests of liver function were unremarkable. Abdominal computed tomography done several months earlier showed small pseudocysts of the pancreas and probable portal vein thrombosis (Figure 10A). Digital subtraction arterial portography revealed cavernous transformation of the portal vein (Figure 10B) and massive splenomegaly but failed to visualize adequately the splenic vein. Splenoportography disclosed a patent splenic vein but with blockage at the superior mesenteric-portal venous juncture and gastric venous collaterization derived almost exclusively from the lesser splanchnic (gastrosplenic) system (Figure 10C). Splenic pulp pressure was 36cm saline. At operation the spleen was markedly enlarged, the liver was grossly normal (light microscopy showed only mild fatty metamorphosis and rare Mallory-Weiss bodies) and there were huge gastro-fundic varices arising from the splenic hilum. The pancreas was diffusely firm consistent with chronic pancreatitis. The spleen was removed, large spleno-fundic venous

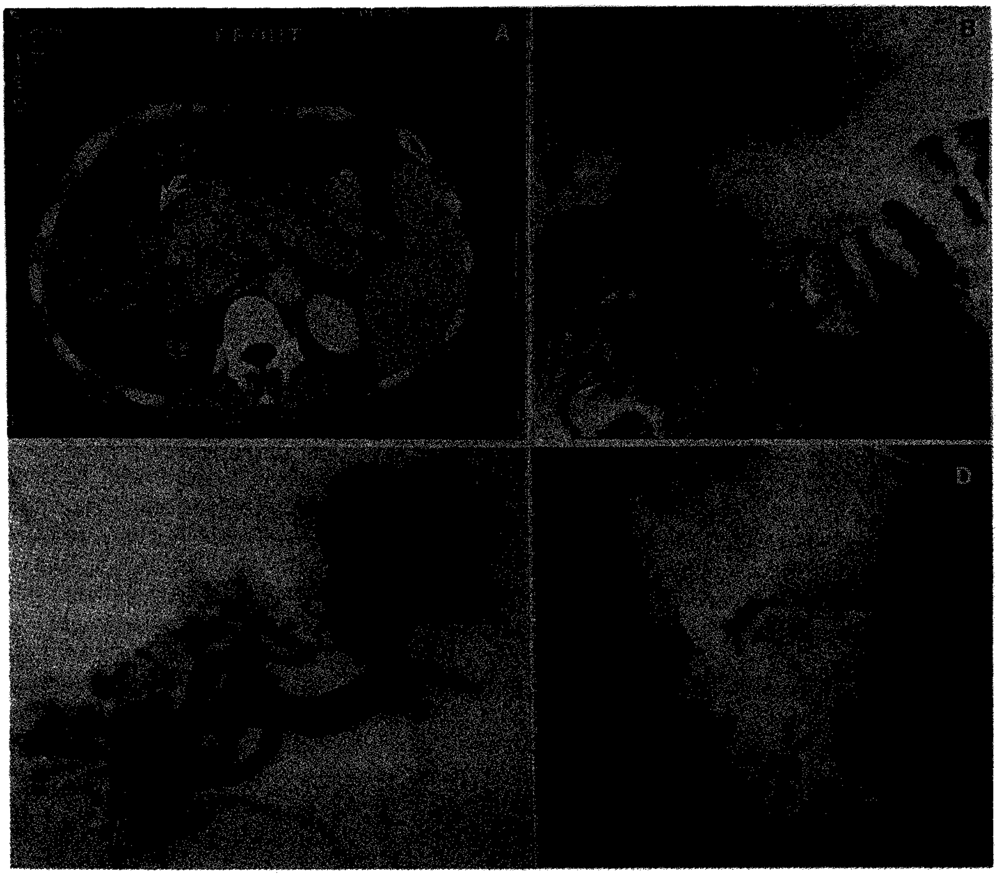

**Figure 10.** Abdominal computed tomography (A) demonstrating multiple small pancreatic pseudocysts and thrombosis of the portal vein (arrow). Digital-subtraction arterial portography confirmed cavernous transformation of the portal vein (B) while splenoportography (C) showed central blockage of the splenic vein and gastric varices arising from the lesser splanchnic system. After splenectomy and independent stapling of the anterior and posterior gastric wall near the fundus, intraoperative portography confirmed cavernous transformation but no esophagogastric varices arising from the greater splanchnic system (D).

collaterals were oversewn and after a small gastrotomy on the greater curvature the anterior and posterior gastric walls were stapled independently (TA-90) approximately 4–6cm distal to the esophagogastric juncture. Intraoperative cannulation of a jejunal mesenteric vein yielded a venous pressure of 27cm and portal venography confirmed occlusion of the superior mesenteric and portal veins but recanalization of flow into the liver (cavernous transformation) (Figure 10D). Specifically there were no major esophagogastric varices arising from the greater splanchnic venous system. Postoperatively, peripheral blood counts including platelets rapidly "normalized" and 23 months later he is well without digestive tract bleeding.

### DISCUSSION

Both the lesser and greater splanchnic portal systems participate in the pathogenesis of esophagogastric varices and their disruption. Normally, the bulk (~75–80%) of portal system blood derives from the mesenteric circulation and a smaller fraction (~20%) from the spleen. In portal hypertension these relative contributions may change. For example, in cirrhosis with splenomegaly, the splenic contribution has been estimated at 40% of splanchnic blood flow[10] but without concomitant measurement of the magnitude of intestinal blood flow, such estimates are potentially misleading. Indeed, recent studies using Doppler pulse sensors and duplex ultrasonography in patients[11,12], and microsphere disappearance kinetics in experimental animals[13,14] suggest that portal hypertension is also associated with augmented superior mesenteric inflow. Portal hypertension, therefore, is a heterogeneous syndrome of divergent clinical manifestations and splanchnic hemodynamics[15,16] much as congestive heart failure involves widely varying systemic and pulmonary circulatory perturbations.

One such subgroup of patients with portal hypertension is described here in whom the splenic circulation is a prominent contributor to the clinical manifestations. Splenomegaly far out of proportion to that accounted for by "passive congestion" is a key feature and derives from both accelerated arterial inflow as well as increased resistance to splenic venous outflow (i.e., "active" rather than "passive" congestion)[17]. In 10 of these patients, the spleen weight after removal was greater than 850gm and alcoholic cirrhosis was an etiologic factor in only two. In another two patients the spleen was also massively enlarged when compared to patient body size. Thus, in these last two patients where the spleen weighed just under 600gms, body weight was only 20kg and 44kg respectively. In other words, in 12 of the 12 patients where spleen weight was measured, the spleen represented more than 1.25% of total body weight (normal 0.25–0.3%) compared with the typical splenomegaly of Laennec's cirrhosis where the spleen characteristically enlarges to only 0.6–0.7% of body weight (~450–500gms).

Besides massive splenomegaly these patients also characteristically displayed huge splenic venous collaterals especially along the greater curvature of the stomach (vasa brevia tributaries), often in conjunction with a large spontaneous fundic variceal-left renal vein shunt. In 8 patients, esophagogastric venous collaterals originated exclusively from the splenic circulation whereas in 5 mesenteric-portal (coronary) collaterals were also demonstrable. Only after both preoperative visceral arterial portography and intraoperative portal venography failed to show coronary venous collaterals was splenectomy alone undertaken. Otherwise, total or near total splenectomy was combined with coronary vein ligation, and the latter corroborated by intraoperative portography. Where these principles were assiduously followed, variceal bleeding either was corrected indefinitely or did not develop when treatment was primarily directed toward relief of anorexia, early satiety, or ascites. Devascularizing operations have previously been proposed[18-23] but seldom using the clinical criteria as we have outlined here. Instead, these operations have been carried out previously on unselected patients, where in many, the spleen was only a minor factor in variceal bleeding; moreover, intraoperative portal venography was not done to verify that the connecting veins to esophagogastric varices were in fact interrupted. Failure to obliterate this porta-azygos pathway may contribute to early varix rebleeding. The addition of proximal gastric wall autostapling may also further reduce the likelihood of recollateralization through the stomach wall.

Despite the removal of a massively enlarged spleen with its attendant hyperdynamic blood flow, in 5 patients portal pressure was minimally affected. Undoubtedly, increased resistance to transhepatic portal flow persisted (i.e., cirrhosis, nodular regenerative hyperplasia or extrahepatic portal vein block) but with unchanged resistance and diminished inflow, one would anticipate a consistent drop in portal pressure. Perhaps, recent demonstration that removal of the spleen in similar circumstances is accompanied by a further rise in already hyperdynamic mesenteric flow accounts for this paradox[11].

In a few patients, portal pressure did fall after splenectomy. Since ascites is primarily a disorder of maldistributed extracellular fluid with deranged salt and water homeostasis and an imbalance in splanchnic lymph formation and absorption secondary to elevated portal microvascular pressure[24], a sharp decline in portal venous pressure after splenectomy was appropriately accompanied by alleviation of ascites as well as varix hemmorrhage. In one patient with repeated episodes of hematemesis from large gastric varices, portal pressure was only slightly elevated, a finding which, however, conforms to observations that variceal bleeding correlates primarily with varix size in accordance with LaPlace's law[25,26]. Thus, rupture of thin-walled varices depends on wall bursting tension, which is a function of vessel diameter and wall thickness as well as intraluminal pressure [25,26].

Even though removal of the spleen is technically simpler than shunt operations (although still treacherous in these patients with giant hilar varices), it is not innocuous as shown by two patients who died later of fulminant pneumococcemia and one of disseminated herpes zoster infection. Overwhelming postsplenectomy sepsis is a bona fide risk in patients with liver disease[27] and supports the use of soluble pneumococcal vaccine and perhaps prophylactic antibiotic drugs, or alternatively preservation of a small splenic remnant. The sequela of disseminated herpes zoster is less clear as patients with immunodeficiency syndromes such as chronic myelogenous leukemia are prone to this viral infection independent of splenectomy[28]. Nonetheless, the unusual severity after removal of the spleen conforms to that seen in patients with myeloproliferative syndromes and an enlarged but immunodefective spleen.[29].

It must be emphasized that this report does not purport to show that splenectomy with, where appropriate, coronary vein ligation is better treatment than portacaval shunt, distal splenorenal shunt, or for that matter transendoscopic sclerotherapy. What it does demonstrate, however, is that there is a subpopulation of patients in whom the abnormal lesser splanchnic system is an important contributor to complications of portal hypertension, and, further, that this subgroup may be managed by an operation less complicated than a portasystemic shunt. Moreover, splenectomy in conjunction with coronary vein ligation does not preclude subsequent transendoscopic sclerotherapy. Indeed, if sclerotherapy has legitimate value, it should work more effectively when major esophagogastric venous feeders from the lesser and greater splanchnic systems have already been interrupted (via splenectomy and coronary vein ligation respectively). In this context, it is noteworthy that Del Guercio and Berman *et al* report [30,31] an analogous tri-pronged approach to bleeding varices (combining transcatheter embolism of the spleen and transhepatic thromboembolism of the coronary venous system with transendoscopic sclerosis).

A major criticism of non-shunt operations for variceal bleeding has been an unacceptably high rebleeding rate albeit not necessarily decreased survival[32]. On the other hand, too little attention is given to when bleeding actually recurs. For example, many patients who have bled from esophagogastric varices fail to survive five years with or without standard shunt operations (perhaps liver transplantation will change this dismal prognosis)[33]. Accordingly, in a select subgroup of patients with massive splenomegaly and large gastro-fundic varices associated with hyperdynamic splenic blood flow often with spontaneous variceal decompression via a retroperitoneal-renal shunt, splenectomy alone may be sufficient for long-term correction of bleeding. Where this constellation of findings is accompanied by greater splanchnic (coronary) collaterals removal of the spleen must also be combined with interruption of these mesenteric venous channels. Moreover, this ligation must be corroborated by intraoperative portal venography (see schema, Figure 11). Whereas progression of the underlying disease (e.g. hepatic cirrhosis and ongoing alcoholism or chronic active viral hepatitis) may further compromise transhepatic portal flow with time and culminate in neocollateralization to the

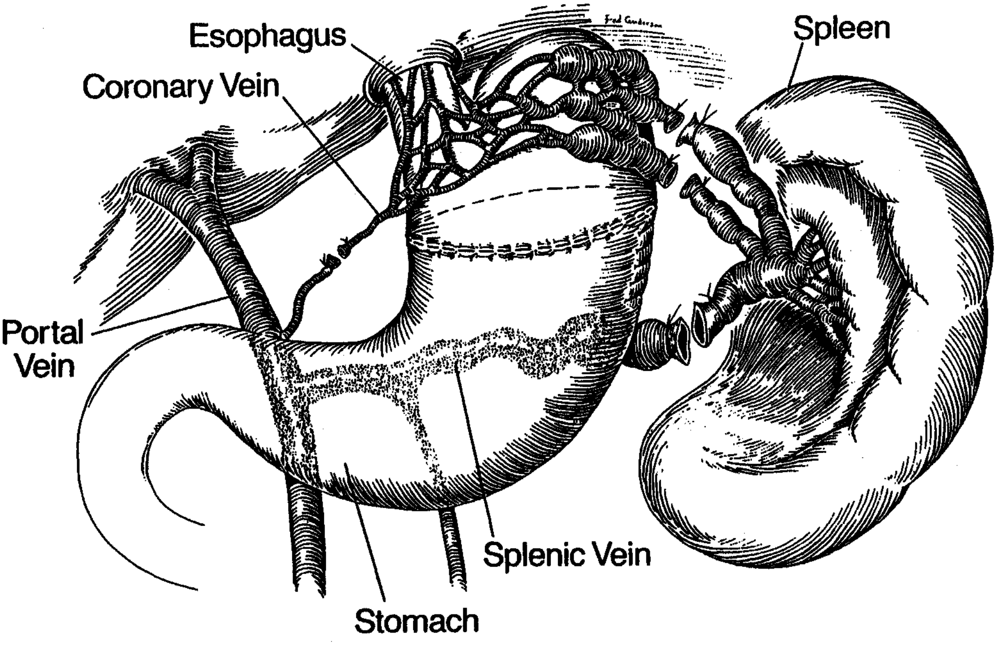

**Figure 11.** Schematic operation for selected patients with generalized portal hypertension in whom the lesser splanchnic (gastrosplenic) circulation is a decisive contributor to clinical complications. These patients are characterized by massive splenomegaly, huge spleno-gastro-fundic varices, and often a spontaneous fundic-left renal venous shunt (not depicted). Where greater splanchnic (coronary) venous collaterals coexist, these must be ligated in addition to splenectomy and the interruption confirmed by intraoperative portal venography (see text). Automatic stapling (TA-90) of the anterior and posterior wall of the stomach separately via a small gastrotomy is optional but may further interrupt transmural portal-azygos venous communications. If necessary, small residual or newly formed esophageal varices can later be obliterated via transendoscopic sclerotherapy.

esophagogastric junction and recurrence of bleeding, the non-shunt operation under the conditions described favors an indefinite remission of variceal bleeding, avoids the drawbacks of a diverted portal stream, and does not preclude and indeed may enhance the efficacy of transendoscopic sclerotherapy by removing large intraperitoneal truncal feeder-veins.

### *References*

1. Gitlin, N., Grahame, G.R., Kreel, L., Williams, H.S., Sherlock, S. (1970) Splenic blood flow and resistance in patients with cirrhosis before and after portacaval anastomoses. *Gastroenterology* **59**: 208–13.
2. Keagy, B.A., Schwartz, J.A., Johnson, G.Jr. (1986) Should ablative operations be used for bleeding esophageal varices? *Ann. Surg.* **203**: 463–69.
3. Salam, A.A., Warren, W.D., Tyras, D.H. (1973) Splenic vein thrombosis: A diagnosable and curable form of portal hypertension. *Surgery* **74**: 961–72.
4. El-Kisher, M.A., Henderson, J.M., Millikan, W.J., Kutner, M.H., Warren, W.D. (1985) Splenectomy is contraindicated for thrombocytopenia secondary to portal hypertension. *Surg. Gyn. Obst.* **160**: 233–237.
5. Leibowitz, H.R. (1959) Bleeding esophageal varices: portal hypertension. Springfield, Illinois: CC Thomas, 866.
6. Witte, C.L., Witte, M.H. (1975) The circulation in portal hypertension. *Yale U Biol. and Med.* **48**: 141–55.
7. Witte, C.L., Witte, M.H., Krone, C.L. (1972) Contrasting hemodynamic patterns of portal hypertension. *Ann. Surg.* **176**: 68–79.
8. Witte, M.H., Witte, C.L., Cole, W.R., Koehler, P.R. (1969)Contrasting patterns of ascites formation in hepatic cirrhosis. *J. Am. Med. Assoc.* **208**: 1661–66.
9. Witte, M.H., Witte, C.L. (1974) Portal hypertension five years after splenectomy. *J. Am. Med. Assoc.* **227**: 1260.
10. Rousselot, L.M. (1949) Combined (one-stage) splenectomy and portocaval shunts in portal hypertension. *J. Am. Med. Assoc* **140**: 282–86.
11. Nishida, O., Moriyasu, F., Nakamura, T., *et al.* (1987) Interrelationship between splenic and superior mesenteric venous circulation manifested by transient splenic arterial occlusion using a balloon catheter. *Hepatology* **7**: 442–46.
12. Sato, S., Ohnishi, K., Sugita, S., Okuda, K. (1987) Splenic artery and superior mesenteric artery blood flow: Non-surgical doppler US measurement in healthy subjects and patients with chronic liver disease. *Radiology* **164**: 347–52.
13. Vorobioff, J., Bredfeldt, J.E., Groszmann, R.J. (1983) Hypernamic circulation in portal-hypertensive rat model: A primary factor for maintenance of chronic portal hypertension. *Am. J. Physiol* **224**: G52–G57.
14. Vorobioff, J., Bredfeldt, J.E., Groszmann, R.J. (1984) Increased blood flow through the portal system in cirrhotic rats. *Gastroenterology* **87**: 1120–26.
15. Witte, C.L., Witte, M.H. (1983) Splanchnic circulatory and tissue fluid dynamics in portal hypertension. *Federation Proc* **42**: 1685–89.
16. Moriyasu, F., Nishida, O., Bon, N., *et al.* (1986) Measurement of portal vascular resistance in patients with portal hypertension. *Gastroenterology* **90**: 710–17.
17. Witte, C.L., Witte, M.H., Renert, W., Corrigan, J.J. Jr. (1974) Splenic circulatory dynamics in congestive splenomegaly. *Gastroenterology* **67**: 498–505.
18. Smith, G.W. (1970) Splenectomy and coronary vein ligation for the control of bleeding esophageal varices. *Am. J. Surg.* **119**: 122.
19. Bothe, A. Jr, Stone, M.D., McDermott, W.V. Jr. (1985) Porta-azygos disconnection for bleeding esophageal varices. *Am. J. Surgery* **149**: 546–50.
20. Tanner, N.C. (1961) Direct operations in the treatment of complications of portal hypertension. *J. Int. Coll. Surg.* **36**: 308–14.
21. Manny, J., Luttwak, E.M., Riukind, A., Eyal, Z. (1985) A simplified one stage modification of portoazygos disconnection for massive variceal hemorrhage. *Surg. Gyn. Obst.* **160**: 171–72.
22. Chung, R.S., Camera, D.S. (1984) Simplified portaazygos disconnection (Tanner's operation) combined with operative sclerotherapy for bleeding varices. *Am. J. Surg.* **148**: 389–92.
23. Romero-Torres, R. (1981) Hemostatic suture of the stomach for the treatment of massive hemorrhage due to esophageal varices. *Surg. Gyn. Ostet.* **153**: 710–12.

24. Witte, C.L., Witte, M.H., Dumont, A.E. (1980) Lymph imbalance in the genesis and perpetuation of the ascites syndrome in hepatic cirrhosis. *Gastroenterology* **78**: 1059–68.
25. Lebrec, D., DeFleury, P., Rueff, B., Nahum, H., Benhamou, J.P. (1980) Portal hypertension, size of esophageal varices, and risks of gastrointestinal bleeding in alcoholic cirrhosis. *Gastroenterology* **79**: 1139–44.
26. Garcia-Tsao, G., Groszmann, R.J., Fisher, R.L., *et al.* (1985) Portal pressure, presence of gastroesophagel varices and variceal bleeding. *Hepatology* **5**: 419–24.
27. Singer, D.B. (1973) Postsplenectomy sepsis. In: Rosenberg, H.S., Bolanger, R.P, eds. Perspectives in pediatric pathology. Chicago, Illinois: *Year Book Medical*, 285–305.
28. Rusthoven, J.J., Ahlgren, P., Elhakim, T., *et al.* (1988) Varicella-zoster infection in adult cancer patients. *Arch. Int. Med.* **148**: 1561–66.
29. Reichman, R.C., Mazur, M.H., Whiteley, R.J., Dolin, R. (moderator). (1978) Herpes zoster-varicellar infections in immunosuppressed patients. *Ann. Intern. Med.* **89**: 375.
30. Del Guercio, L.R.M., Hodgsin, W.J., Morgan, J.C., Berman, H.L., Kinkhabovilla, M.N. (1984) Splenic artery and coronary vein occlusion for bleed esophageal varices. *World J. Surg.* **8**: 680–87.
31. Berman, H.L., Del Guercio, L.R.M., Katz, S.G., Hodgson, W.J., Savino, J.A. (1988) Minimally invasive devascularization for variceal bleeding that could not be controlled with sclerotherapy. *Surgery* **104**: 500–06.
32. Umeyama, K., Yoshikawa, K., Yamashita, T., Todo, T., Satake, K. (1983) Transabdominal oesophageal transection for oesophageal varices: experience in 101 patients. *Brit. J. Surg* **70** 419–22.
33. Iwatsuki, S., Starzl, T.E., Todo, S., *et al.* (1988) Liver transplantation in the treatment of bleeding esophageal varices. *Surgery* **104**: 697–705.

### INVITED COMMENTARY

In this report, Dr. Witte and his colleagues have collected a series of 13 patients with generalized portal hypertension associated with predominantly gastric rather than esophageal varices. These patients were treated by splenectomy and coronary vein ligation. Their case studies and pertinent angiographic studies are also included. Porto-mesenteric pressures recorded during the operation were evaluated and consistent with generalized portal hypertension, distinguishing these patients from those with isolated splenic vein thrombosis or "sinistral portal hypertension". The subsequent clinical course of the patients was used to support a "forward flow" explanation of portal hypertension, i.e. that increased splenic inflow, is primarily responsible for variceal bleeding in their group. The authors were careful to state that these patients were selected to illustrate their point from a larger series of variceal bleeders treated by other means. Although the cause of liver disease in the group was quite heterogenous, a common finding was massive splenomegaly and hypertrophid short gastric veins. Of interest is that few of the patients were alcoholics with Laennec's cirrhosis and the degree of splenomegaly seen in this series was much greater than is usually found in portal hypertensive alcoholics. Only one of the patients rebled during follow-up. The authors conclude from their observations that there is a subgroup of patients with portal hypertension in whom the splenic component of flow is responsible for variceal bleeding and these patients can be identified by a massively enlarged spleen with hypertrophied short gastric veins. In such patients splenectomy is suggested as a surgical approach to treatment.

Investigation into the role of the spleen in portal hypertension has a long and venerable history. In the early part of this century, splenopneumopexy (transfering the spleen into the thorax) was considered one approach to managing variceal bleeding. Banti hypothesized that splenic inflow was responsible for variceal bleeding in some patients with noncirrhotic liver disease. It was in the famous "Spleen Clinic" at Columbia University that Dr. A. O. Whipple popularized portacaval and splenorenal shunts for varix hemorrhage in the 1940's. In the 1950's and 60's, Womack and his colleagues at the University of North Carolina investigated splenectomy and proximal gastrectomy for portal hypertension. Modifications, including gastric stapling and devascularization with splenectomy have been used successful in Japan. However, when nonshunt procedures, including splenectomy, have been applied to patients with portal hypertension, mostly alcoholic patients in the US, the results have been less than satisfactory, with about 50% rebleeding rates.

The concept of sinistral or left-sided portal hypertension associated with splenic vein thrombosis, pancreatitis or tumor compression of the splenic vein has also been well-described. In such patients, splenectomy is curative. Unlike the patients presented in this report, patients with isolated splenic vein thrombosis have normal livers and normal portal pressures. What makes this report intriguing is that perhaps splenectomy can also be applied to selected patients with generalized portal hypertension avoiding the necessity of a shunt operation with its attendant morbidity.

While I appreciate that the author is trying to make a point about the splenic contribution to portal flow, if the value of this operation for variceal bleeding is being studied, I believe that the evidence should be limited to patients with this complication. Of the 13 patient histories presented, only 8 had bleeding varices. Of these, 2 were postoperative deaths and 3 more were followed for less than 1 year. Thus only 3 patients with varices were folllowed for longer than a year. It would also be interesting to know how many patients with variceal bleeding were in the larger group that this series of patients was drawn from. Is massive splenomegaly and hypertrophied gastric varices a common or uncommon finding? Also, splenectomy limited the surgeon's options by preventing the subsequent use of distal splenorenal shunt.

The above comments notwithstanding, the authors' conclusions may in fact be valid and certainly deserve further investigations. We and others have recently encountered patients bleeding actively from gastric varices following successful obliteration of esophageal varices with endoscopic sclerotherapy. A few have continued to bleed for more than 24 hours following totally decompressive portacaval shunts. If the authors' hypothesis is correct, splenectomy may be an appropriate way to manage these patients.

Eric B. Rypins
Associate Professor of Surgery
Department of Surgery
California College of Medicine
University of California Irvine Medical Center
101 The City Drive
Orange; California 92668–9969

